# Diverse transcriptional regulation and functional effects revealed by CRISPR/Cas9-directed epigenetic editing

**DOI:** 10.18632/oncotarget.28037

**Published:** 2021-08-17

**Authors:** Miguel Vizoso, Jacco van Rheenen

**Affiliations:** ^1^Division of Molecular Pathology, Oncode Institute, The Netherlands Cancer Institute, Amsterdam, The Netherlands

**Keywords:** targeted DNA methylation, CRISPR/Cas9-based system, *IGFBP2*, epithelial-to-mesenchymal transition

## Abstract

DNA methylation is an epigenetic process that controls DNA accessibility and serves as a transcriptomic switch when deposited at regulatory regions. The adequate functioning of this process is indispensable for tissue homeostasis and cell fate determination. Conversely, altered DNA methylation patterns result in abnormal gene transcription profiles that contribute to tumor initiation and progression. However, whether the consequence of DNA methylation on gene expression and cell fate is uniform regardless of the cell type or state could so far not been tested due to the lack of technologies to target DNA methylation *in-situ*. Here, we have taken advantage of CRISPR/dCas9 technology adapted for epigenetic editing through site-specific targeting of DNA methylation to characterize the transcriptional changes of the candidate gene and the functional effects on cell fate in different tumor settings. As a proof-of-concept, we were able to induce *de-novo* site-specific methylation of the gene promoter of *IGFBP2* up to 90% with long-term and bona-fide inheritance by daughter cells. Strikingly, this modification led to opposing expression profiles of the target gene in different cancer cell models and affected the expression of mesenchymal genes *CDH1*, *VIM1*, *TGFB1* and apoptotic marker *BCL2*. Moreover, methylation-induced changes in expression profiles was also accompanied by a phenotypic switch in cell migration and cell morphology. We conclude that in different cell types the consequence of DNA methylation on gene expression and cell fate can be completely different.

## INTRODUCTION

Increasing number of studies report that proteins do not work in isolation but are part of a complex network of biomolecules, that may differ at various settings (e.g., different tumor types [1, 2] or stages of tumor progression [3]). Various examples of genes with opposing roles, e.g., during tumorigenesis, have been described in literature [4–7]. DNA methylation may be one of the drivers of these opposing roles. For example, the same DNA methylation mark can lead to very opposite outcomes (embryonic viability versus lethality) depending on which allele is tagged with this modification [8]. Here we will test whether the same epigenetic modification could also orchestrate molecular and phenotypic diversity in non-imprinted genes.

In this study, we will focus on the insulin-like growth factor binding protein 2 (*IGFBP2*), a recently discovered multitasked gene regulated by DNA methylation which has also been reported to function both as a tumor-promoting and -suppressing gene. IGFBP2 is a secreted protein that competes with IGF-1 and IGF-2 ligands for IGF receptor binding, thereby modulating the downstream cascade of IGF signaling that mediates essential cellular processes such as proliferation and migration. On one hand, it has been described as a tumor suppressor by promoting a p53-dependent *IGF-1* and *ERK* inactivation and therefore leading to proliferation attenuation [9]. Additionally, in cooperation with TGFB1, SERPINE1, and BCL2 it has been shown to induce apoptosis and block migration [10]. On the other hand, *IGFBP2* has been shown to act as an oncogene since it promotes invasion through interaction with integrin α5 and β1 [11, 12], it activates the NFkβ-Zeb1 [13] and EGFR/STAT3 axes [14], it promotes vascular mimicry by CD144 and MMP2 [15], and it induces immunosuppression [16].

The diverse phenotypes upon single alterations in cancer driver genes may simply reflect the various roles of these genes in distinct signaling pathways, but it may also be caused by differential gene expression patterns of these genes. For example, the hormone estradiol upregulates the expression levels of *IGFBP2* in MCF7 cells whilst in R3230AC mammary adenocarcinoma it leads its downregulation [17–19]. However, it is unknown whether other regulatory mechanisms of gene expression, such as DNA methylation [20], can also lead to opposing expression patterns in different cell types when the levels of DNA methylation remain constant (similarly to the hormone situation). DNA methylation comprises a significant mechanism involved in transcriptomic regulation based on the occupancy of CpG dinucleotides by 5’-methylcytosine chemical groups [21–23]. Whether the consequence of DNA methylation on the expression of the target genes and cell fate is uniform regardless of the cell type or can drive to opposing phenotypes depending on the tumor cell context is still unknown, and it has remained elusive for many years due to the lack of technologies to target DNA methylation *in-situ*.

Here, we take advantage of the CRISPR/Cas epigenome editing technology [24], and evaluate the contribution of this *de-novo* epigenetic modification to *IGFBP2* transcriptomic regulation and cell plasticity. We report the first CRISPR/dCas9 epigenetic editing of the *IGFBP2* promoter showing long-term and bona-fide inheritance of DNA methylation by daughter cells. Our study also highlights that comparable increments of DNA methylation can lead to opposing transcription effects on the expression of the target gene in different tumor cell types and leads to dysregulation of mesenchymal genes and one apoptotic marker. Moreover, we show that DNA methylation can induce a phenotypic switch in migration and cell morphology.

## RESULTS

### Gene-specific DNA methylation by CRISPR/dCas9/DNMT3ACD module

**Figure 1:**
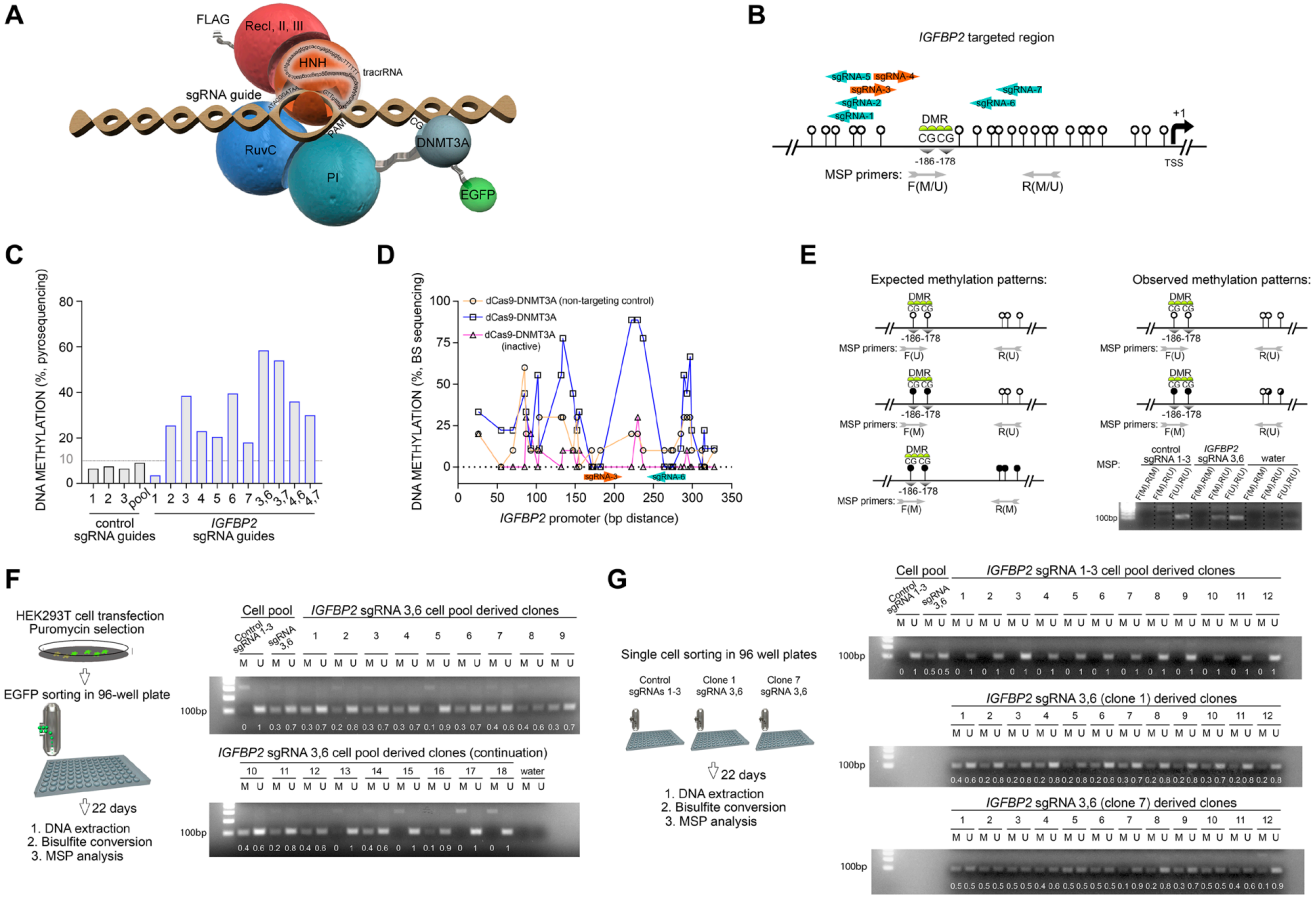
RNA-programmed DNA methylation of *IGFBP2* promoter introduces stable and heritable marks across mitotic cell divisions. (**A**) Predicted structure of the dCas9 protein fused with the DNMT3ACD and DNA-anchoring guided by the sgRNA sequence. The recognition lobe (RecI, II and III domains) and the nuclease lobe (HNH, RuvC and PI domains) of Cas9 protein are also represented. (**B**) Location of the sgRNA guides in the promoter region of the *IGFBP2* gene. The two CpG sites interrogated are denoted as differential methylated region (DMR) and placed according to their distance to the transcription start site (TSS). White lollipops represent adjacent CpG sites. Methyl-specific primer (MSP) location. F/R(M), forward/reverse primer for methylated region; F/R(U), forward/reverse primer for unmethylated region. (**C**) Pyrosequencing analysis for the evaluation of DNA methylation in HEK293T cells comparing cells targeted with control or *IGFBP2*-specific guides. Single and pooled sgRNA guides were tested. (**D**) Increase in CpG methylation level in the *IGFBP2* promoter region targeted by either pooled control sgRNAs/dCas9-DNMT3A (active), specific sgRNAs/dCas9-DNMT3A (active), or specific sgRNAs/dCas9-DNMT3A (inactive) constructs. (**E**) Schematic representation of the expected and observed DNA methylation patterns after CRISPR/dCas epigenetic editing and pyrosequencing. Confirmation of pyrosequencing results by MSP (amplicon size 102 bp). F/R(M), forward/reverse primer for methylated region; F/R(U), forward/reverse primer for unmethylated region. (**F**) Strategy followed to isolate individual cells from the transfected population for the study of DNA methylation stability. MSP PCR amplifications obtained from cells transfected with a pool of control sgRNA guides 1-3 (gel lanes 1 and 2); a pool of *IGFBP2* specific sgRNA guides 3 and 6 (gel lanes 3 and 4); and 18 single clones derived from the targeted cell population (sgRNAs 3,6) after 22 days of cell culture. Gel band densitometries are indicated. (**G**) Long-term DNA methylation analysis performed on 12 single control clones (derived from the pooled sgRNAs 1-3 cell population) and 12 single *IGFBP2* targeted clones derived from Figure 1G clones 1 and 7. Lanes 1-2 (control pooled sgRNAs 1-3) and 3-4 (*IGFBP2* targeted pooled sgRNAs 3,6) represent the negative and positive controls for DNA methylation, respectively. Gel band densitometries are indicated.

To target specific DNA methylation sites *in-vitro*, we used the inactive Cas9 endonuclease (dCas9, the targeting domain, p.D9A and p.H840A) fused to DNMT3ACD. The predicted tethering of the fusion protein to the genomic DNA is depicted in Figure 1A. Two nuclear localization sequences were placed upstream and downstream of the Cas9 sequence to promote an enrichment of the fusion protein into the nucleus. To select positive cells expressing the dCas9-DNMT3ACD construct, eGFP fluorescence sequence was coupled to the 3’-terminus of the DNMT3ACD using a T2A linker. First, we validated our system by targeting the exact differential methylation regions (DMRs) reported by Vojta et al. (2016) regarding the *BACH2* gene (Supplementary Figure 1A). Pyrosequencing confirmed the same average levels of DNA methylation in DMR1 as previously reported (Supplementary Figure 1B). Moreover, some individual CpG sites of the DMR2 reached peaks of DNA methylation close to the reported values (e.g., CpG1, 6% when using control sgRNAs and 15% when targeting *BACH2* promoter with sgRNA 3). This data shows that our approach to target specific methylation sites works to a comparable extent as it has been reported previously for a similar approach [25].

### DNA methylation of *IGFBP2* promoter is stable across mitotic cell divisions

Using our validated system, we evaluated the transiently-performed *de-novo* targeted DNA methylation of *IGFBP2* promoter and its stable inheritance across several rounds of mitotic cell divisions. We selected seven sgRNA sequences targeting the *IGFBP2* promoter CpG site 1 and 2 (Figure 1B). DNA methylation was targeted in this specific region which represents the binding site of p53, one of the transcription factors involved in *IGFBP2* gene expression. HEK293T cells were transfected with the dCas9-DNMT3ACD construct and its expression was evaluated on a protein level detecting a band by western blot at the expected size (210 kDa) using an anti-flag antibody (Supplementary Figure 2A). To target DNA methylation, HEK293T cells were transfected with dCas9-DNMT3ACD construct and the corresponding sgRNA guides. Five days post transfection dCas9-DNMT3ACD-positive cells were selected based on their eGFP expression (Supplementary Figure 2B). Pyrosequencing revealed that the sgRNAs located within a window of 27–30 bp upstream or downstream of the targeted CpG sites rendered the higher increments of DNA methylation in comparison to the control sgRNAs (Figure 1C). Particularly, single sgRNAs 3 and 6 managed the higher individual increments of DNA methylation in the two targeted CpGs (up to 40%). Interestingly, the combination of sgRNAs 3 and 6 or 3 and 7, which target the region of interest in a head-to-head orientation, exhibited the highest DNA methylation efficiencies (up to 60%), demonstrating that the combination of sgRNAs is crucial for more efficient targeting (Figure 1C). Bisulfite sequencing performed on the same genomic locus validated the pyrosequencing data and revealed a clear gain of DNA methylation when the active form of the DNMT3A enzyme is tethered to the targeted region (Figure 1D, Supplementary Figure 3A). Additionally, and confirming previously reported observations [25], bisulfite sequencing showed peaks of DNA methylation occurring on the flanking regions of the dCas9 binding, at 27–30 bp distance from the PAM sequence (Figure 1D, Supplementary Figure 3A).

To measure the stability of DNA methylation inheritance, we set up the methyl-specific primer (MSP) assay for our region of interest. This technique permits to analyze a high number of samples with minimum costs in comparison with pyrosequencing approach. First, MSP tests confirmed that DNA methylation is not gained in the distal downstream CpG sites of the targeted region, but instead in the interrogated CpG sites (Figure 1E), supporting the pyrosequencing data. In order to quantify methylation, we made use of the fact that the downstream CpG sites remained always unmethylated, and carried out all MSP PCR amplifications using an unmethylated-specific reverse primer. When non-specific sgRNAs were transfected, the interrogated CpG sites remained unmethylated, whilst the pooled sgRNAs 3 and 6 provided a MSP PCR amplification pattern in accordance with the DNA methylation levels detected by pyrosequencing and bisulfite sequencing (Figure 1E).

To determine the inheritance of the *de-novo* epigenetic mark, both pooled control sgRNAs 1–3 and sgRNAs 3 and 6 cell populations were eGFP sorted, single cells were isolated and clonal lines were derived (Figure 1F). After 22 days and hence several rounds of cell divisions, clonal MSP PCR amplifications showed that most of the single clones targeted with pooled sgRNAs 3 and 6 remained methylated (77.8%, 14 out of 18 clones) (Figure 1F). Bisulfite sequencing performed on two randomly selected clones validated the MSP results confirming the hypermethylation of the targeted region after 22 days in culture (Supplementary Figure 3B). Additionally, in order to further characterize the long-term inheritance of this targeted epigenetic mark, two randomly selected CRISPR/dCas IGFBP2 targeted clonal populations (1 and 7) were expanded and seeded again as singles clones by sorting. After another 22 days of expansion, the analysis of 12 derived clones per condition indicated that DNA methylation was fully retained in most of the the interrogated clonal populations targeted with sgRNAs 3 and 6 (Figure 1G). Bisulfite sequencing confirmed the full long-term retention of DNA methylation in one of the randomly selected clones (88%) and a partial retention in a second clone after 44 days in culture (25%, Supplementary Figure 3C). Combined, our data show that the CRISPR/dCas-DNMT3ACD system facilitates specific promoter methylation of the *IGFBP2* gene with a 60–89% efficiency and that its retention lasted several round of population doublings in the actively dividing HEK293T cells.

### Target DNA methylation on *IGFBP2* promoter modifies mRNA levels

**Figure 2:**
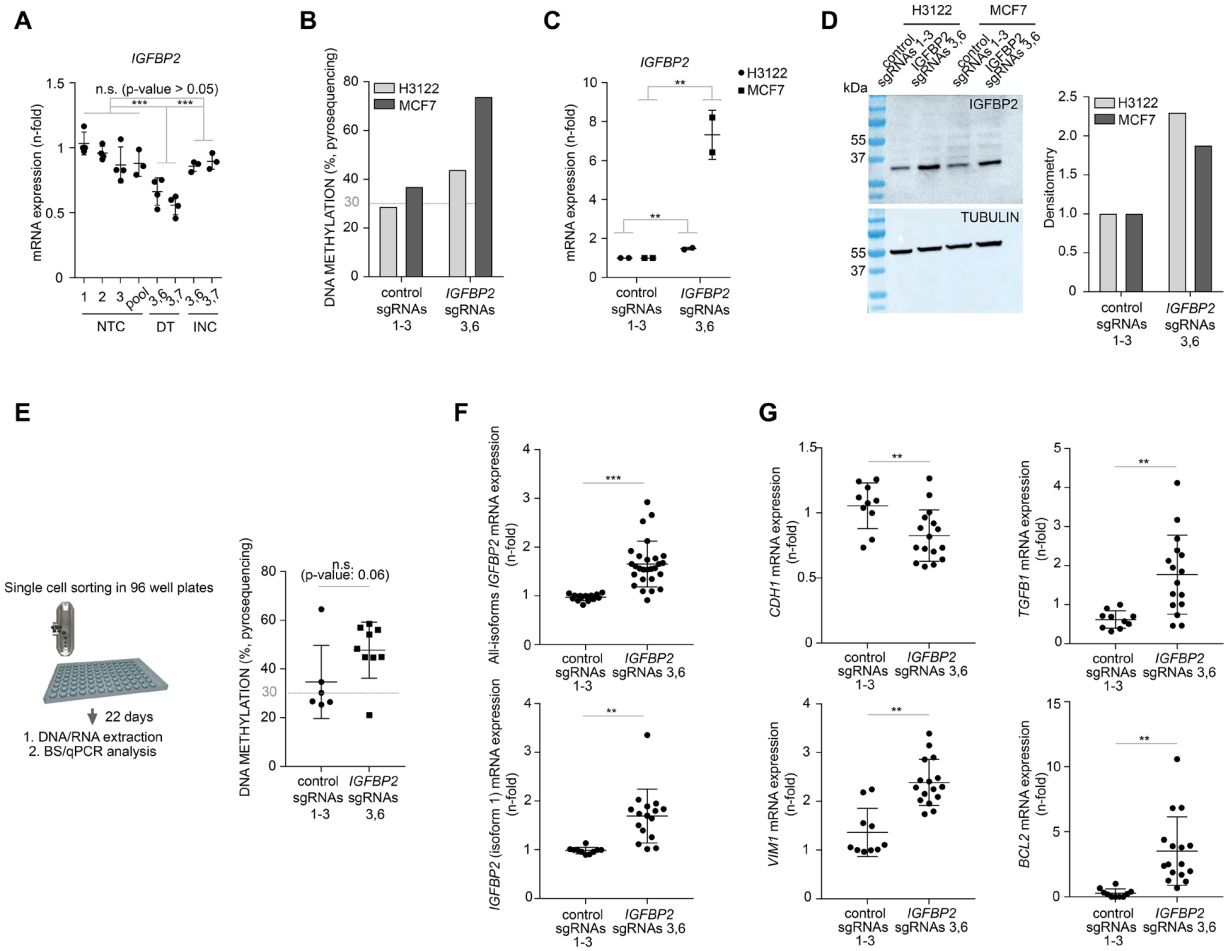
CRISPR/dCas targeted DNA methylation of *IGFBP2* promoter affects gene transcription and EMT transcriptomics in cancer cells. (**A**) qPCR analysis to evaluate the expression of *IGFBP2* gene in HEK293T cells. NTC: non-targeting control, DT: direct targeting, INC: DNMT3A inactive control. Comparisons are stablished between 3-4 independent biological replicates [including control populations (both single or pooled control sgRNAs 1–3) and 2 independent *IGFBP2* targeted populations (sgRNAs 3,6 and 3,7)]. Data is normalized using first control and housekeeping genes *B2M* and *PPIA* (error bars represent SD). Individual *p*-values were obtained from Tukey’s multiple comparison test. (**B**) Pyrosequencing analysis for the evaluation of DNA methylation in H3122 and MCF7 cells comparing cells targeted with control or *IGFBP2*-specific guides 3 and 6. (**C**) qPCR analysis to evaluate the expression of *IGFBP2* in H3122 and MCF7 cells. Data is normalized using first control and housekeeping gene *B2M* (error bars represent SD of two independent biological replicates). (**D**) Western blot and densitometry showing the expression of *IGFBP2* after CRISPR/dCas editing in H3122 and MCF7 cells. (**E**) Left panel, strategy followed to isolate individual clones from MCF7 cells transfected with pooled control sgRNAs 1-3 or *IGFBP2*-specific guides 3 and 6. All derived clones from each condition were evaluated for the levels of DNA methylation by pyrosequencing (right panel). *P*-value was obtained using Mann-Whitney test. (**F**) The expression of *IGFBP2* all-isoforms (upper panel) and *IGFBP2* isoform 1 (lower panel) was evaluated for all single clones from each condition. Statistics were performed comparing 2 independent biological replicates [including 7 independent control clones (pooled control sgRNAs 1–3) and 10 independent *IGFBP2* targeted clones (sgRNAs 3,6)]. Data was normalized using first control and housekeeping genes *B2M* and *PPIA* (error bars represent SD). (**G**) qPCR analysis to evaluate the expression of mesenchymal genes (*CDH1*, *VIM1*, *TGFB1*) and apoptotic marker *BCL2* in MCF7 clones from Figure 2F. Comparisons are stablished using data from two independent biological replicates (including 7 independent single clones transfected with pooled control sgRNAs 1–3 and 10 independent single clones transfected with *IGFBP2*-specific sgRNAs 3 and 6). Data is normalized using first control and housekeeping genes *B2M* and *PPIA* (error bars represent SD). qPCR statistical analysis were performed using one sample *T*-test after log2 data transformation. Two–tailed *p*-values ≤ 0.05, ≤ 0.01, or ≤ 0.001 are considered statistically significant and indicated by an asterisk (^*^, ^**^, or ^***^, respectively).

After showing that *IGFBP2* promoter is epigenetically editable, we next questioned if this could have any impact on the transcriptional levels. We compared four control conditions based on three single non-targeting control sgRNAs (used independently or as a pool), and two different combination of targeting sgRNAs (sgRNAs 3 and 6; and sgRNAs 3 and 7). As expected, by increasing the levels of methylation, we observed a significant reduction in the transcriptional levels of *IGFBP2* gene when the active DNMT3A construct was specifically targeted into this locus (Figure 2A). The methylation of *IGFBP2* promoter did not led to the transcriptional dysregulation of EMT regulatory genes *CDH1*, *VIM1* or *TGFB1*. However, we found a significant differential expression of the apoptotic marker *BCL2* depending on the levels of methylation of *IGFBP2* promoter (Supplementary Figure 4A). In conclusion, in HEK293T cells, targeted DNA methylation on *IGFBP2* promoter affects its own transcription and the levels of expression of *BCL2* apoptotic regulatory gene.

### Gain of DNA methylation of *IGFBP2* promoter increases expression levels of mesenchymal-like genes in epithelial-like tumor cells

In order to study the effect of targeting *IGFBP2* promoter DNA methylation on cell plasticity, we proceeded by conducting similar experiments in two epithelial tumor cells lines (H3122 and MCF7). H3122 is a lung adenocarcinoma cell line and MCF7 cells represent a breast cancer cell line. First, we achieved a significant increase of DNA methylation of 43.8% and 73.7% in H3122 and MCF7 cell lines, respectively (Figure 2B). Bisulfite sequencing validated these results with levels of methylation of 50% and 86% in H3122 and MCF7, respectively (Supplementary Figure 4B and 4C). This epigenetic editing led to a significant dysregulation in the transcriptional levels of *IGFBP2* gene. Surprisingly, opposite to what we observed in HEK293T cells, in these epithelial tumor models there was a significant upregulation of the mRNA and protein levels upon CRISPR/dCas targeting with locus-specific sgRNAs (Figure 2C and 2D).

To follow up on this surprising observation, which contradicts the canonical view that DNA methylation on gene promoters downregulate gene expression, we repeated the epigenetic editing using a new batch of MCF7 cells. In these cells we included the inactive DNMT3A construct as an additional control. First, we noticed that the new batch of cells showed a remarkable low basal level of DNA methylation in the *IGFBP2* locus compared to the old batch (0% vs 22%, Supplementary Figures 4B and 5A), highlighting epigenetic divergence within the same cell type. By targeting the *IGFBP2* locus with the active DNMT3A and unspecific guides or the inactive DNMT3A and locus-specific guides, we observed a modest gain of background methylation (38%), comparable to the old batch. However, direct targeting using the active construct and locus-specific guides managed an increase of DNA methylation up to 69% (Supplementary Figure 5A). This indicates that the locus is epigenetically editable in the new batch of cells but also reveals that the gain of DNA methylation is almost 20% less efficient than using the old batch of cells. As we initially expected, mRNA expression data revealed a non-significant but consistent downregulation of *IGFBP2* upon epigenetic editing with the active DNMT3A in the new MCF7 batch (Supplementary Figure 5B). Importantly, cell check validations identified all the cell lines and batches used in this study and confirmed their suspected origin (Supplementary Figure 5C). Overall, our data suggests that the basal levels of DNA methylation and the efficiency of CRISPR/dCas9/DNMT3A targeting could determine the downstream transcriptional effects.

Intrigued by our observation of *IGFBP2* upregulation by DNA hypermethylation in two independent tumor cells lines (H3122 and MCF7), we continued exploring in greater detail the old MCF7 batch which showed the highest *IGFBP2* mRNA upregulation. First, we took a closer look into the *IGFBP2* mRNA isoforms. The *IGFBP2* gene contains four mRNA isoforms but only isoform 1 (NM_000597) overlaps with the targeted CpG island. Additionally, to reveal if cell heterogeneity was responsible for these unexpected results, we performed the analysis on 7 (pooled sgRNAs 1–3) and 10 (pooled *IGFBP2* sgRNAs 3 and 6) MCF7 single cell clones. Pyrosequencing analysis of individual clones confirmed a clear DNA methylation enrichment when the promoter region of *IGFBP2* was targeted by CRISPR/dcas9/DNMT3A (Figure 2E). Importantly, qPCR data revealed that the increase in DNA methylation was specifically associated with the upregulation of *IGFBP2* isoform 1 (the more prevalent isoform) in most of the clones tested (Figure 2F). Isoforms 2, 3 and 4 were also analyzed but the expression levels were very low or even absent and did not differ between clones (data not shown). Therefore, the differences in mRNA expression levels between control and targeted clones were mainly explained by the expression of *IGFBP2* isoform 1.

Taking advantage of the reported evidences that *IGFBP2* can affect the transcriptional levels of EMT [26] and apoptotic markers [10], we interrogated the single clones derived from the MCF7 cell line and could confirm the significant impact of this epigenetic modification on the transcriptional levels of EMT and apoptotic related genes upon *IGFBP2* hypermethylation. *IGFBP2* hypermethylation led to the downregulation of *CDH1* and upregulation of *VIM1* and *TGFB1* genes (Figure 2G). Additionally, *IGFBP2* hypermethylation also induced the upregulation of *BCL2* gene (Figure 2G).

**Figure 3:**
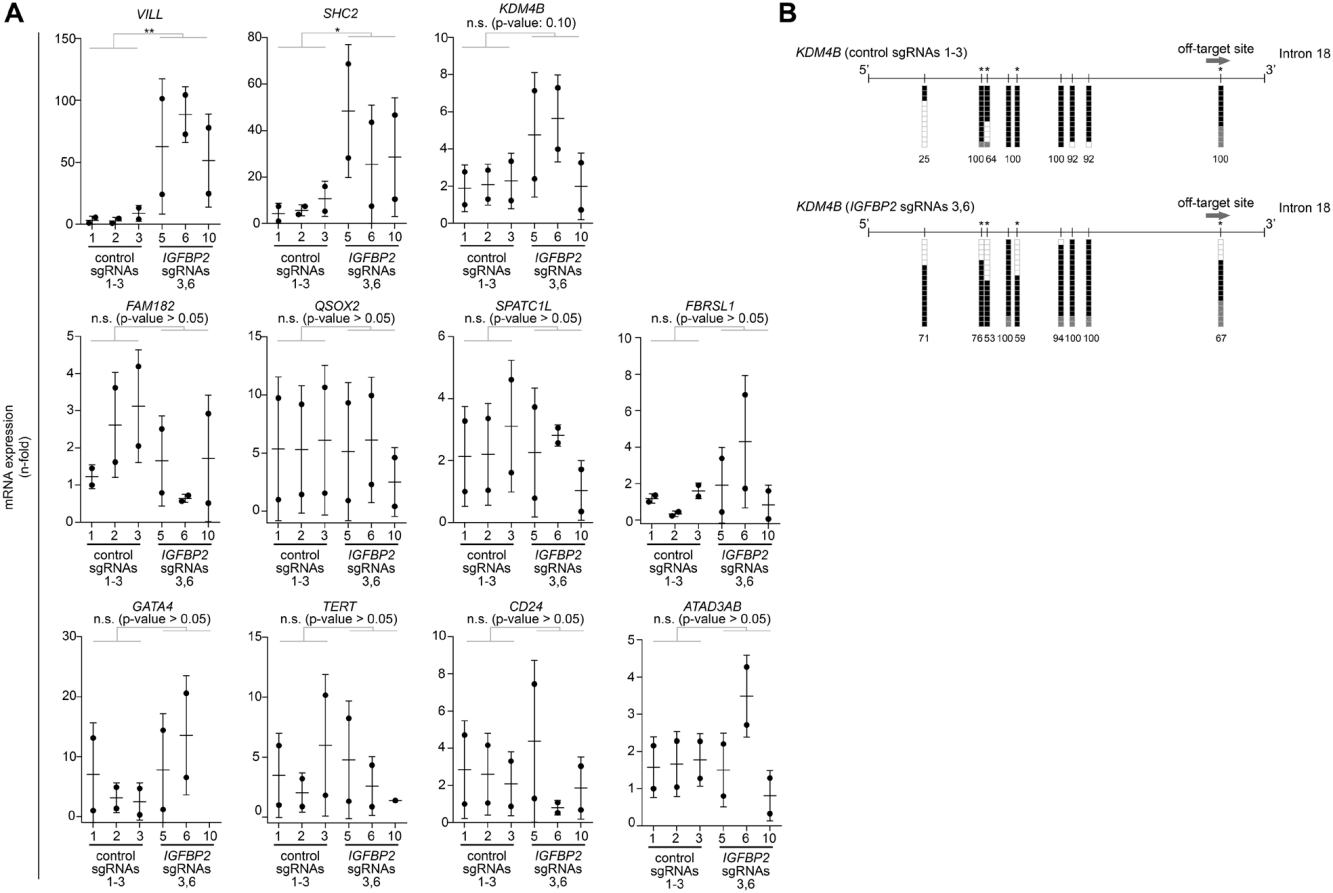
Off-target analysis identified some indirect effects by CRISPR/dCas targeting. (**A**) qPCR analysis to evaluate the expression of CRISPR/dCas off-target genes for *IGFBP2* sgRNA guides 3 and 6. Comparisons are stablished between 2 independent biological replicates [including 3 independent control clones (transfected with pooled control sgRNAs 1-3) and 3 independent *IGFBP2* targeted clones (transfected with sgRNAs 3 and 6)]. Data is normalized using first control and housekeeping genes *B2M* and *PPIA* (error bars represent SD). Statistical analyses were performed using one sample *t*-test after log2 data transformation. Two–tailed *p*-values ≤ 0.05, ≤ 0.01, or ≤ 0.001 are considered statistically significant and indicated by an asterisk (^*^, ^**^, or ^***^, respectively). (**B**) Bisulfite sequencing of the off-target candidate region linked to *KDM4B* gene. Each vertical line denotes a CpG site within the interrogated region. Bisulfite genomic sequencing was carried out in ≥ 12 individual clones. The presence of a methylated or unmethylated cytosine is indicated by a black or white square, respectively. Gray squares denote CpG sites with unconverted cytosines outside the CpG region so they are not considered for the estimation of the DNA methylation (denoted by numbers, %). Vertical lines highlighted with an asterisk represent CpG sites with a reduction in DNA methylation.

To test also for CRISPR/dCas unspecific binding effects, we performed an off-target gene analysis of the most commonly-used guides in this study (sgRNAs 3 and 6). Most of the off-target candidates (*FAM184B*, *QSOX2*, *SPATC1L*, *FBRSL1*, *GATA4*, *TERT*, *CD24*, *ATAD3AB*) did not show any difference in expression between the targeted and non-targeted clones (Figure 3A). However, our CRISPR/dCas epigenetic editing clearly upregulated some of the top-ranked off-target genes (*VILL*, *SHC2*, *KDM4B*) (Figure 3A). To test whether this upregulation of those genes could be a consequence of an off-targeted methylation, we performed bisulfite sequencing on one of the off-target regions belonging to the histone demethylase KDM4B/JMJD2B. Surprisingly, this analysis revealed that DNA methylation levels in 4 out of the 9 CpGs of the off-target region of *KDM4B/JMJD2B* gene (located at 3’ end of intron 18) was decreased (Figure 3B). Overall, the transcription levels of 2 out of the 13 off-target candidates (15%) were significantly changed and bisulfite sequencing data obtained in one of the candidate regions suggests this could be directly orchestrated by an unspecific CRISPR/dCas binding.

### DNA methylation of *IGFBP2* promoter alters cell morphology and increases migration velocity in MCF7 cells

**Figure 4:**
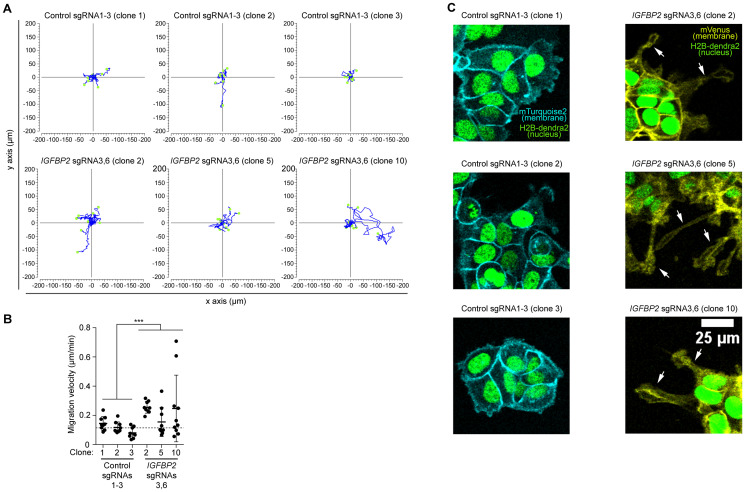
High resolution imaging reveal an increased migration speed of the epithelial cell line MCF7 clones upon CRISPR/dCas *IGFBP2* epigenetic editing. (**A**) Display of 9 independent migratory tracks from 3 pooled control sgRNAs 1-3 clones and 3 pooled *IGFBP2* sgRNAs 3 and 6 clones. (**B**) Distribution of migration velocity from 3 pooled control sgRNAs 1-3 clones and 3 pooled *IGFBP2* sgRNAs 3 and 6 clones. Error bars represent the SD of *n* = 3–9 independent positions. Statistical analysis were performed using Mann-Whitney *U* test for speed and displacement and *T*-test for mitotic cell division analysis. *p* values ≤ 0.05, ≤ 0.01, or ≤ 0.001 are considered statistically significant and indicated by an asterisk (^*^, ^**^, or ^***^, respectively). (**C**) Representative time-lapse images of 3 pooled control sgRNAs 1-3 clones and 3 pooled *IGFBP2* sgRNAs 3 and 6 clones. White arrow indicates the appearance of cell protrusions (absent in the control conditions). Images show nuclear marker (H2B-dendra2) to follow cell divisions and migration and membrane markers (mTurquoise-2 and mVenus) to study cell morphology.

Since *IGFBP2* promoter DNA methylation leads to alteration of expression in genes related with migration, we tested whether it has functional consequences for this process. Therefore, we set up time-lapse live imaging and characterized migration behavior of the *IGBFP2*-epigenetically edited epithelial cancer cell line MCF7 in comparison to their controls. In this experiment, three pooled sgRNAs 1–3 control clones and three *IGFBP2* pooled sgRNAs 3 and 6 clones were tested. We imaged the cells for 14 hours, and manually tracked the position of each cell. As depicted in the rose plots in Figure 4A and 4B, the *IGFBP2* targeted clones migrated significantly faster than the control clones. A detailed analysis of the cell surface highlighted that all *IGFBP2* targeted clones experienced a cell morphology transformation with a significant increase in the number and length of cell protrusions (Figure 4C and Supplementary Videos 1–4, white arrows). Overall, these results, in line with the transcriptomic data, support the idea that the epigenetic editing of *IGFBP2* gene drives a more migratory phenotype in the breast epithelial cancer cell line MCF7.

## DISCUSSION

In this study we revealed the consequence of DNA methylation on the expression of *IGFBP2*, and we additionally gain some basic insights into on the process of DNA methylation itself. The precise editing of the *IGFBP2* promoter locus by CRISPR/dCas/DNMT3ACD technology revealed that the combination of head-to-head oriented sgRNAs targeting adjacent sites of the region of interest enabled methylation at higher levels. Our data also indicates that the maximum peak of DNA methylation is reached at 27–30 bp distance from the PAM sequence and that the methylation efficiency starts to drop after 35 bp, confirming previous results [25, 27]. Moreover, we provide evidence that targeted methylation of DNA, introduced by CRISPR/dCas epigenetic editing, is inherited by daughter cells and stable across multiple cell divisions. To our knowledge, no study has reported to date whether transiently induced CRISPR/dCas edited DNA methylation is stably transmitted from parental-to-daughter cells by performing clonal experiments. Several publications have previously reported that single DNA methylation changes introduced by CRISPR/dCas-DNMT3ACD technology into the mammalian genomes were quickly lost, suggesting the presence of cell counteracting mechanisms to retain the cell specific DNA methylation fingerprint [25, 27]. Here, we showed a substantial stability of DNA methylation transmission from parental-to-daughter cells by studying the clonal inheritance of this epigenetic mark throughout many rounds of mitotic cell divisions. Our results suggest that the edited epigenetic marks are stably kept in the genome of daughter cells for more than 48 cell divisions that occur within more than a month in cell culture. Interestingly, these results line up with the most recent publication describing the programmed long-term inheritance of *de-novo* DNA methylation by the transient use of the CRISPRoff system [28]. However, we cannot rule out that in other genomic regions rather than promoters, like repetitive element sequences or intergenic regions, retention of ectopic DNA methylation marks could follow different dynamics. In fact, recent reports showed that promoter regions are better preserved in terms of DNA methylation and that non-functional regions, such as those containing repetitive elements, show a higher (epi)variance [29].

Our study also provides new insights into the regulation of *IGFBP2* expression. *IGFBP2* is a multitasked protein with extracellular and intracellular functions. This oncogene was originally described as a direct target for p53-mediated transcription, blocking the activation of phosphor-ERK expression and therefore inhibiting IGF-I signaling [9]. More recently, many studies identified *IGFBP2* as an EMT-driver gene that promotes the proliferation and migration of colorectal cancer cells through E-cadherin inhibition [30] or the hepatocellular carcinoma progression by activation of the NF-κB-ZEB1 signaling axis [13]. In gliobastomas, *IGFBP2* affects a myriad of different molecular networks and shaping tumor progression by unbalancing the EGFR-STAT3 signaling and potentiating STAT3 phosphorylation [14], connecting integrins and NF-κB signaling to cell migration [31], and promoting tumor cell trans-differentiation into endothelial cells (vasculogenic mimicry) via expression of vascular-endothelial cadherin CD144 and MMP2 [15]. This literature suggests that the expression of this gene must be tightly regulated in order to guarantee its correct functionality. *IGFBP2* levels are regulated by hormones, protease activity, hypoxia and as more recently shown, promoter DNA methylation [32]. Here, we show that *IGFBP2* expression can also be regulated by targeted DNA methylation.

Targeted methylation of promoter regions is thought to lead to decreased expression of those genes [33]. Here, we have studied the consequence of targeted methylation of the *IGFBP2* promoter, and show that methylation of this promoter lead to opposite transcriptional effects of this gene in different cell types and batches. In HEK293T cells, with only 7% of basal DNA methylation, we find a strong and significant negative correlation between *de-novo* DNA methylation in two specific CpG sites of *IGFBP2* promoter and the expression levels of *IGFBP2*. Similar observations were obtained with the new batch of MCF7 cells which shows 0% of basal DNA methylation at the interrogated CpG sites. Surprisingly, in another two cell settings (H3122 and old batch MCF7) we found higher levels of basal DNA methylation (~25%) and a positive correlation between *de-novo* DNA methylation and *IGFBP2* expression both at mRNA and protein levels. Our bisulfite sequencing and expression data indicates that the differential basal levels of DNA methylation of *IGFBP2* locus in the wild type cells correlates with opposite expression outcomes upon epigenetic editing. This suggests that basal levels of methylation can determine the transcription outcomes derived from the epigenetic editing.

Cell clonal analysis of *IGFBP2* mRNA isoforms performed on old batch MCF7 cells confirmed the aforementioned results and additional gene expression measurements revealed a significant positive correlation between the increment of DNA methylation, the downregulation of epithelial marker *CDH1* and the upregulation of EMT genes *VIM1* and *TGFB1*. To our knowledge, this is the first report to demonstrate that the DNA methylation of *IGFBP2* promoter has an impact on the expression levels of mesenchymal-like markers. By means of CRISPR/dCas *IGFBP2* DNA methylation, we observed a significant downregulation of *CDH1* and upregulation of *VIM1* and *TGFB1* expression in all clones interrogated. Moreover, we found that the morphology, migration and therefore growth pattern is severely altered in the epigenetically targeted MCF7 clones. The effects of TGFB1 on growth on estrogen receptor positive cells lines like MCF7 is well-known and is supported by our findings [10]. In the present study, we have also found that DNA methylation, which affects the levels of *IGFBP2* expression, also unbalances *BCL2* expression in two different tumor cell line models (HEK293T and MCF7). Interestingly, these two genes have in common that they belong to two strong anti-apoptotic systems (IGF-1 and the pro-survival BCL2-like proteins) and their reciprocal modulation has been reported before [34].

In conclusion, our study highlights the significance of exploring the effects of the epigenetic editing in different tumor settings by revealing the important consequences that this can have on transcriptomic regulation and tumor cell behavior.

## MATERIALS AND METHODS

### CRISPR/dCas9-DNMT3ACD fusion protein and sgRNAs design

To target specific DNA methylation *in-vitro*, we focused on the system previously described by Vojta et al. (2016). The catalytic domain of human DNMT3A (amino acids P602-V912; herein, DNMT3ACD) fused with the inactive SpCas9 (upstream) and the T2A-eGFP coding sequences (downstream) were obtained from the plasmid pdCas9-DNMT3A-eGFP (Addgene plasmid #71666). Two substitutions in the coding sequence (D10A and H840A) abolished the nickase activity of the endonuclease. The coding sequence starts with a triple flag-tag followed by a SV40 nuclear localization signal. An additional NLS sequence in frame with a short Gly4Ser linker is placed between the dCas9 and DNMT3A sequences. This cassette was digested with the restriction enzymes AgeI and NotI and cloned into Addgene plasmid #63592 previously digested with XbaI and EcoRI, in order to substitute the CMV promoter with the EF-1alpha promoter. For the final ligation two 50–60 bp adaptors were added following overnight incubation using T4 ligase from NEB (ref. M0202). Vector sequence can be found in Supplementary Material 1.

Guide sequences targeting the locus of interest were designed using the Breaking-Cas web-tool [35] (http://bioinfogp.cnb.csic.es/tools/breakingcas). Only 20 nucleotide length guides with NGG PAM sequence, reaching scores above 85 over 100 were selected. Three non-targeting control guide RNAs with no match in the human genome were taken from the Human GeCKOv2 Library [36]. Forward and reverse primers were re-suspended in water to reach 100 μM. Then 2.5 ul of each primer was added to 120 μl of annealing buffer (100 mM NaCl and 50 mM Tris pH 7.5). The PCR program was as follows: 95°C 5 min for 14 cycles (each cycle the temperature was diminished 5°C, until it reached 25°C) and a final step at 25°C for 20 min. sgRNAs are shown in Supplementary Table 1A.

### Cell transfection

Human embryonic kidney cell line HEK293T, H3122, and MFC7 were maintained in Dulbecco’s Modified Eagle Medium (#21885-025, Invitrogen) supplemented with 10% fetal bovine serum, 1% sodium pyruvate (H3122 cells), 100 U/ml penicillin and 100 μg/ml streptomycin. Cells were incubated at 37*°*C in a humidified 5% CO_2_ environment.

For cell transfection, cells were seeded at 80–90% of confluence in 6-well plates and 24 hours later cells were transfected with Lipofectamine 2000 as follows: a mixture of plasmids (3 μg of dCas9-DNMT3A-eGFP plasmid and 1 μg of sgRNA guide plasmid) were mixed in 150 μl of Optimem media. For the cases where combination of guides were needed, we used equimolar amounts of each sgRNA guide never exceeding 1 μg of sgRNA in total. In parallel, 10 μl of Lipofectamine 2000 was diluted in 150 μl of Optimen media and incubated at room temperature for 5 min. Then, the plasmid mixture was added to the lipofectamine dilution and incubated at room temperature for 20 min. Finally, cells were refreshed with 2 ml of pre-warmed DMEM+10% FBS media and the transfection mixture was added drop-wise. After 24 hours, cells were harvested and transferred to a 10 cm dish adding 1.5 μg/ml of puromycin to enrich the population in cells expressing the sgRNA guides. Selection was performed for 48 hours. After that, cell culture was refreshed with media and maintain in culture for two days. Finally, cells were harvested and eGFP-FACS was performed.

### FACS sorting

Sorting of cells was performed in Aria Fusion (BD Biosciences) upon cell resuspension in FACS buffer containing 2 mM of EDTA and 2% of FBS in PBS. A broad FSC/SSC gate was followed by gates excluding doublets and selection of Topro3-living cells. eGFP+ tumor cells were isolated using stringent gating.

### DNA methylation analysis

DNA was extracted from cells by phenol:chloroform method adapted for low number of cells. Briefly, recovered cells from FACS were resuspended in 600 μl of lysis buffer (10 mM Tris-HCl pH7.4, 10 mM EDTA, 200 mM NaCl, 1%SDS) and 15 μl of proteinase K (10 mg/ml). Samples were incubated overnight (ON) at 37*°*C. After ON incubation, proteinase K was inactivated at 75*°*C for 15 min and then 2 μl of RNAse (10 mg/ml) was added. After an incubation of 30 min at 37*°*C, 620 μl of phenol:chloroform:isoamilic (25:24:1) was added and mixed for 10 min in rotation at room temperature. After 30 min centrifugation at 12,000 g (4*°*C), the acuose phase was put in a new tube, mixed with an equal volume of clorophorm for 5 min at room temperature. After 15 min centrifugation at 12,000 g (4*°*C), the acuose phase was put in a new tube and 1/10 volumes of NaAc (3M) was added and mixed. Then, an equal volume of isopropanol and 50 μl/ml GlycoBlue (Ambion, AM9515) were added and mixed for 5 min at room temperature. Samples were kept at –20*°*C ON. Finally, samples were 30 min centrifuged at 12,000 g (4*°*C) and washed twice with ethanol 70%.

Bisulfite conversion of 100–1000 ng of genomic DNA was performed using an EZ DNA methylation kit (Zymo Research) following the manufacturer’s instructions. Genomic DNA was converted using an EZ DNA Methylation Gold kit (Zymo Research, Orange, CA, USA) and Bibikova et al. (2009) denaturation conditions (initial denaturation step at 98*°*C 10 min, and 16 cycles at 98*°*C 30 sec and 50*°*C for 1 h). DNA methylation was studied by pyrosequencing, which was performed on bisulfite-treated DNA extracted from cells. Pyrosequencing reactions and quantification of DNA methylation were performed in a PyroMark Q24 System version 2.0.6 (QIAGEN) including appropriate controls. Specific primers were designed using the PyroMark Assay Design Software (QIAGEN-version 2.0.01.15) for pyrosequencing to examine the methylation status of particular CG sites covering the candidate genes promoter regions. Pyrosequencing primers are shown in Supplementary Table 1B.

For bisulfite sequencing analysis, bisulfite converted DNA was used as a template in a PCR reaction. BS primers are shown in Supplementary Table 1C. The PCR program was run using the Immolase Taq (Bioline) and adjusted to maximize the PCR product amplification without reaching the saturation point: initial denaturation step at 95*°*C for 8 min, 31 cycles (95*°*C 30 sec, 59*°*C 30 sec, 72*°*C 25 sec), and a final extension of 72*°*C 1 min. The 519 bp PCR band was purified from agarose gel and clone into PGEM-T (Promega) vector. Dh5-alpha bacteria colonies containing the insert (white colonies) were selected by IPGT and X-gal and correct integration was confirmed by sequencing.

For MSP analysis, bisulfite converted DNA was used as a template in a PCR reaction. Specific primers for the unmethylated and methylated region were designed using MethylExpress^®^ program (Applied Biosystems). MSP primers are shown in Supplementary Table 1D. The primers contain 2 to 3 CpG sites at the 3’ end of each primer. The PCR program was run using the GoTaq^®^ G2 Hot Start Green Master Mix (Promega, M7421) and adjusted to maximize the PCR product amplification without reaching the saturation point: initial denaturation step at 95*°*C for 8 min, 31 cycles (95*°*C 30 sec, 59*°*C 30 sec, 72*°*C 25 sec), and a final extension of 72*°*C 1 min. This approach permits to analyze a high number of samples with minimum costs in comparison with pyrosequencing.

### Expression analysis

For qRT–PCR experiments, total RNA was extracted using Trizol^®^ reagent and 1 ug was retrotranscribed using the Kit High Capacity cDNA Reverse Transcription Kit (ref. 4368814). Real time PCR was performed using PowerUp SYBR Green (A25741), and PPIA, B2M and TBP were used as housekeeping genes to enable normalization. Primers were used at 150 nM concentration and sequences are annotated in Supplementary Table 1E. RDML raw data was processed using RDML-Ninja and LinReg softwares to determine the baselines and obtain the empirical primer efficiencies. qPCR fold changes between samples were obtained by ΔΔCt calculations and corrected by primer efficiencies. Average values from the two housekeeping genes were calculated, log2 transformed and plotted. Statistical significance was calculated with GraphPad Prism package using one sample *t*-test. For immunoblotting assays, total protein was extracted using Laemli 1X (60 mM Tris-HCl, 2% SDS, 10% glycerol, bromophenol blue 0.01%) to quickly preserve the integrity of all the protein phosphorylation sites. Specific antibodies against target proteins are listed in Supplementary Table 1F.

### Time-lapse confocal imaging

To characterize the dynamic behavior of epigenetically edited cells six single clones of the MCF7 breast cancer model were used (three pooled sgRNAs 1–3 control clones and three IGFBP2 pooled sgRNAs 3,6 clones). For that, each clone was co-transduced with a cell membrane marker (based on the MARCKS domain) and a nuclear marker (based on the H2B-dendra2 reporter). In order to distinguish control clones from IGFBP2 targeted clones, the former were transduced with MARCKS-mTurquoise2 fluorophore and the latter with MARCKS-mVenus fluorophores. Vector sequences can be found in Supplementary Material 1. Time-lapse was performed using a Leica confocal SP5 inverted microscope with 25X water immersion objective (N.A. 0.95) water objective. In brief, 2,000 cells were plate per well in 8-ibidi glass bottom chambers and kept in their own media preventing moisture drift and guarantying CO_2_ supply by using an Okolab CO_2_ chamber. Images were taken every 12 minutes for a total of 14 hours approximately. Images were post-processed using Fiji software. Cell migration track displays, speed, and displacement values were obtained using Chemotaxis and Migration Tool V2.0 (Ibidi) upon retrieving the XY coordinates from Fiji. Statistical analysis were performed with SPSS package using KS and SW normality test and Mann-Whitney *U* tests (for cell migration speed and displacement) and *t*-test for independent samples (for cell division).

### CRISPR/dCas9 off target analysis

CRISPR/dCas9 off target candidates for IGFBP2 sgRNAs 3 and 6 were obtained using the software package Breaking-Cas web-tool [35] (http://bioinfogp.cnb.csic.es/tools/breakingcas). Off-target candidate regions are annotated in Supplementary Table 2A and 2B. Using NCBI browser (GRCh38) we retrieved for each candidate a genomic sequence including 500 bp (upstream and downstream) from the 20 bp off-target sequence. Then, each sequence was individually interrogated for the presence of enriched CpG regions by using the MethylExpress software. Genes linked to the off-target candidate regions associated to (partial) CpG islands were further characterized by qPCR comparing three control pooled sgRNAs 1–3 clones with three of the IGFBP2 pooled sgRNAs 3 and 6 clones with the most significant dysregulation of cell plasticity genes. Primers are listed on Supplementary Table 1D. Bisulfite sequence of the off-target gene candidate KDM4B was additionally performed as previously described. BS primers are listed on Supplementary Table 1C.

## SUPPLEMENTARY MATERIALS














