# Supplementary material for: Diverse transcriptional regulation and functional effects revealed by CRISPR/Cas9-directed epigenetic editing
Source: Oncotarget. 2021 Aug 17;12(17):1651–62. doi: 10.18632/oncotarget.28037 (PMC8378768; doi:10.18632/oncotarget.28037)
Supplement: Supplementary file 1 [file oncotarget-12-1651-s001.pdf]

Diverse transcriptional regulation and functional effects revealed by CRISPR/Cas9-directed epigenetic editing

SUPPLEMENTARY MATERIALS

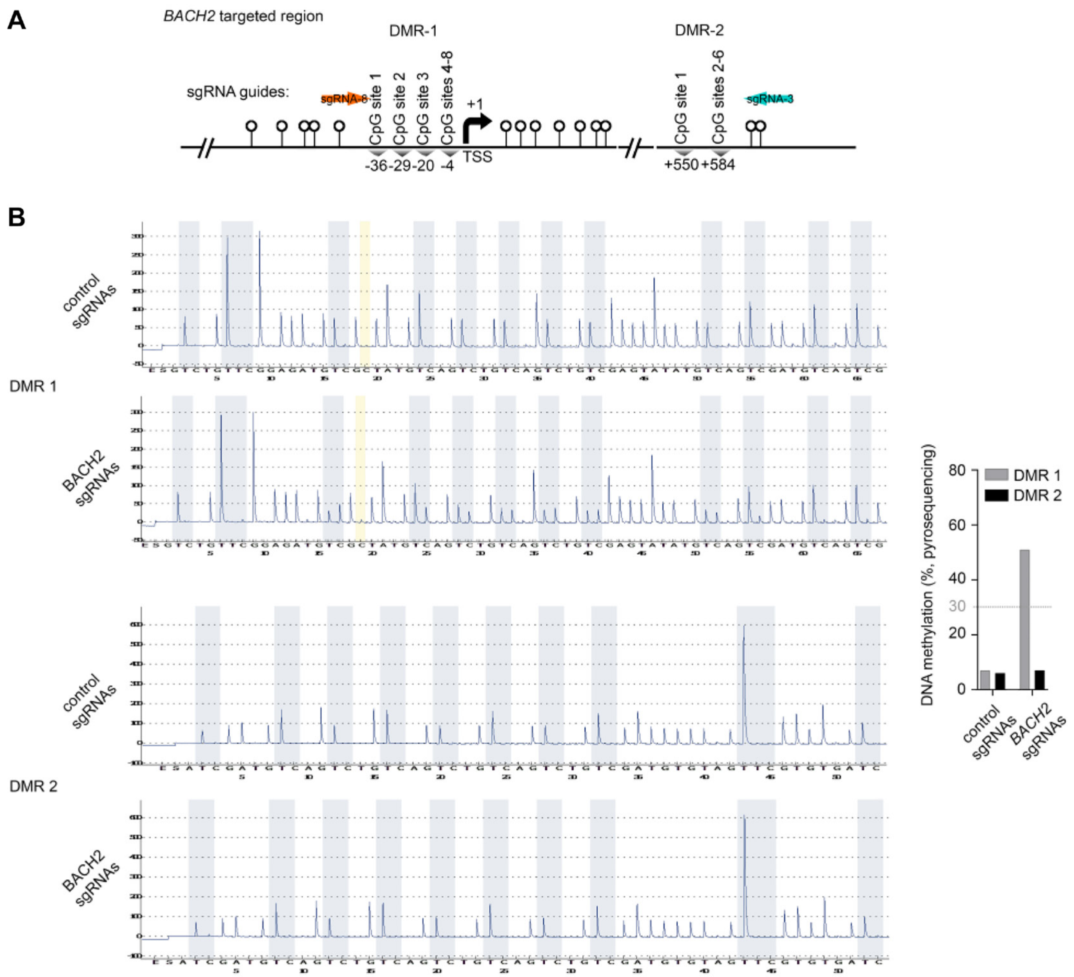

Supplementary Figure 1: Validation of CRISPR/dCas-DNMT3ACD epigenetic editing in *BACH2* gene in HEK293T cells. (**A**) Graphical representation of *BACH2* gene promoter highlighting DMR1 and 2. sgRNAs 3 and 8 matching regions, targeted CpG sites and transcription start site are indicated. White lollipops denote non-targeting CpG sites. (**B**) Pyrosequencing data performed on DMR1 and 2 and quantification.

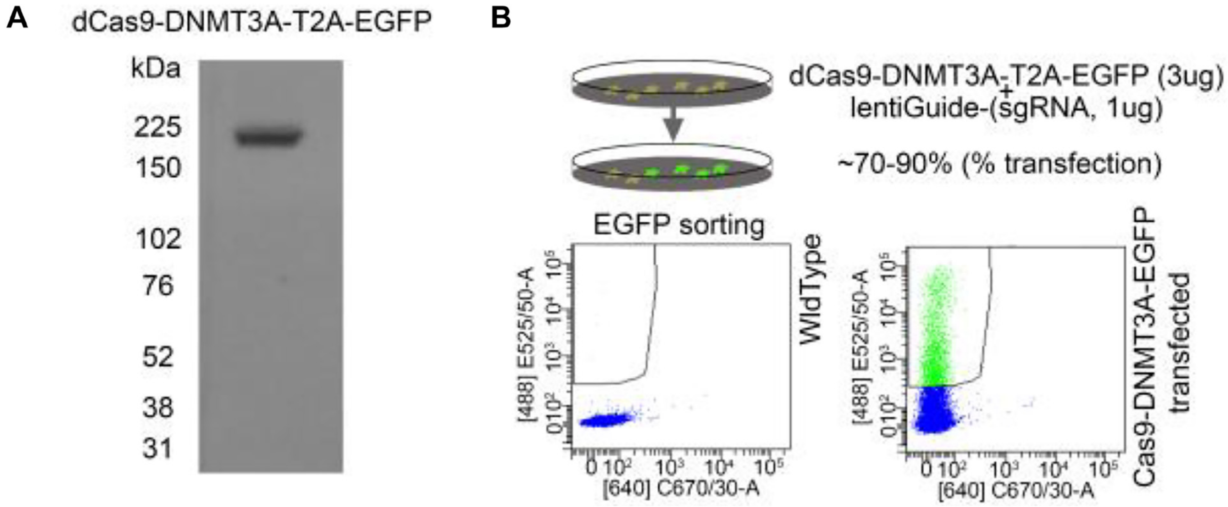

Supplementary Figure 2: Validation of CRISPR/dCas-DNMT3A protein size and FACS strategy. (**A**) Western blot showing the expression of the dCas9-DNMT3A-eGFP fusion protein. (**B**) Strategy followed for the transfection of HEK293T cells with CRISPR/dCas system and FACS analysis.

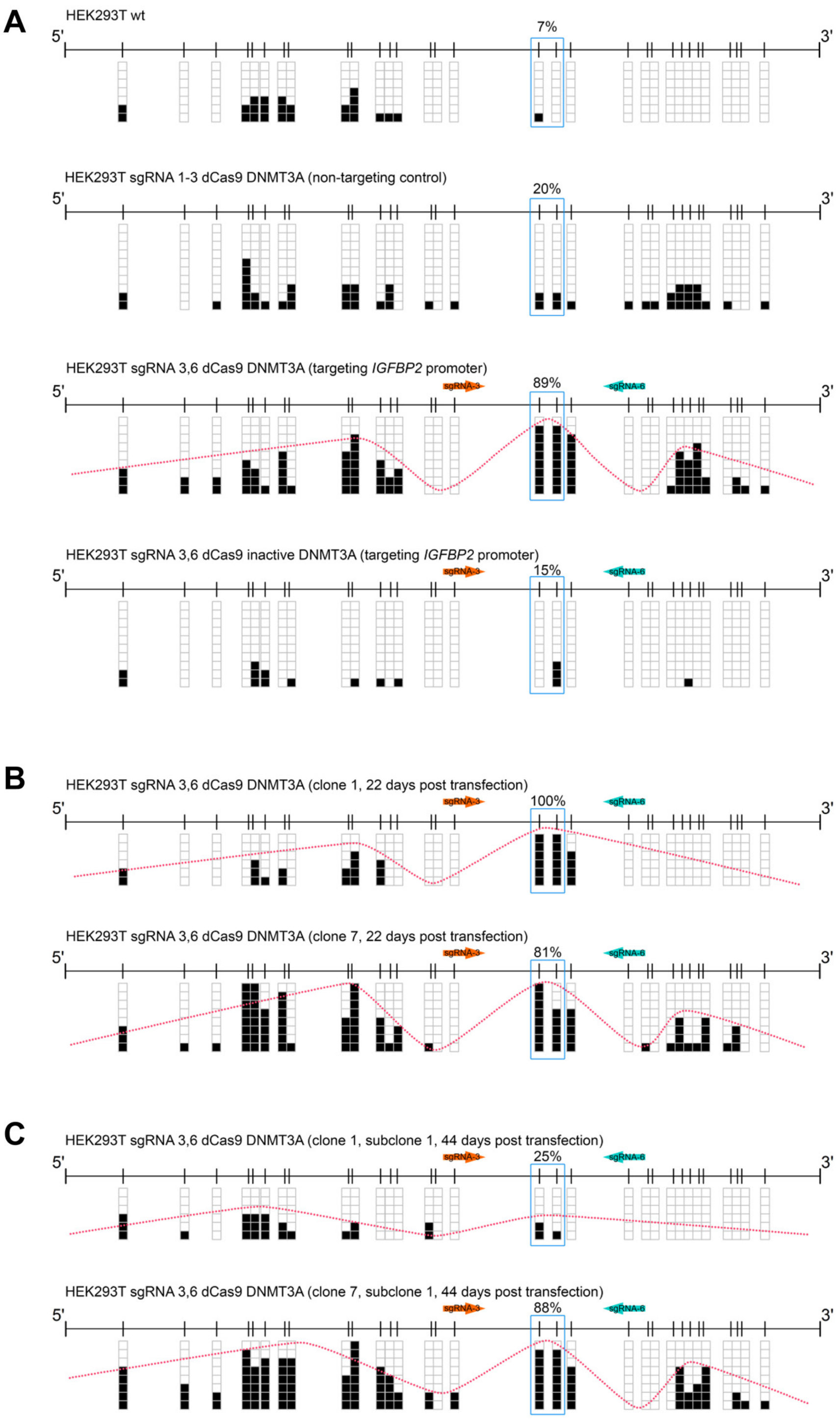

Supplementary Figure 3: (**A**) Bisulfite sequencing of *IGFBP2* locus in native and dCas9/DNMT3A targeted HEK293T cells. (**B**) Bisulfite sequencing of two independent clones isolated from the cell pool population targeted with *IGFBP2* specific sgRNA guides 3,6 and maintained for 22 days in culture. (**C**) Bisulfite sequencing of two independent clones isolated from the two clonal populations represented in (B) and maintained in culture for another 22 days. Single sgRNA guides tethering positions are indicated by colored arrows. Bisulfite genomic sequencing was carried out in 7–8 individual clones. The presence of a methylated or unmethylated cytosine is indicated by a black or white square, respectively. CpGs sites of interest are highlighted with a blue squared and the percentage of methylation is indicated.

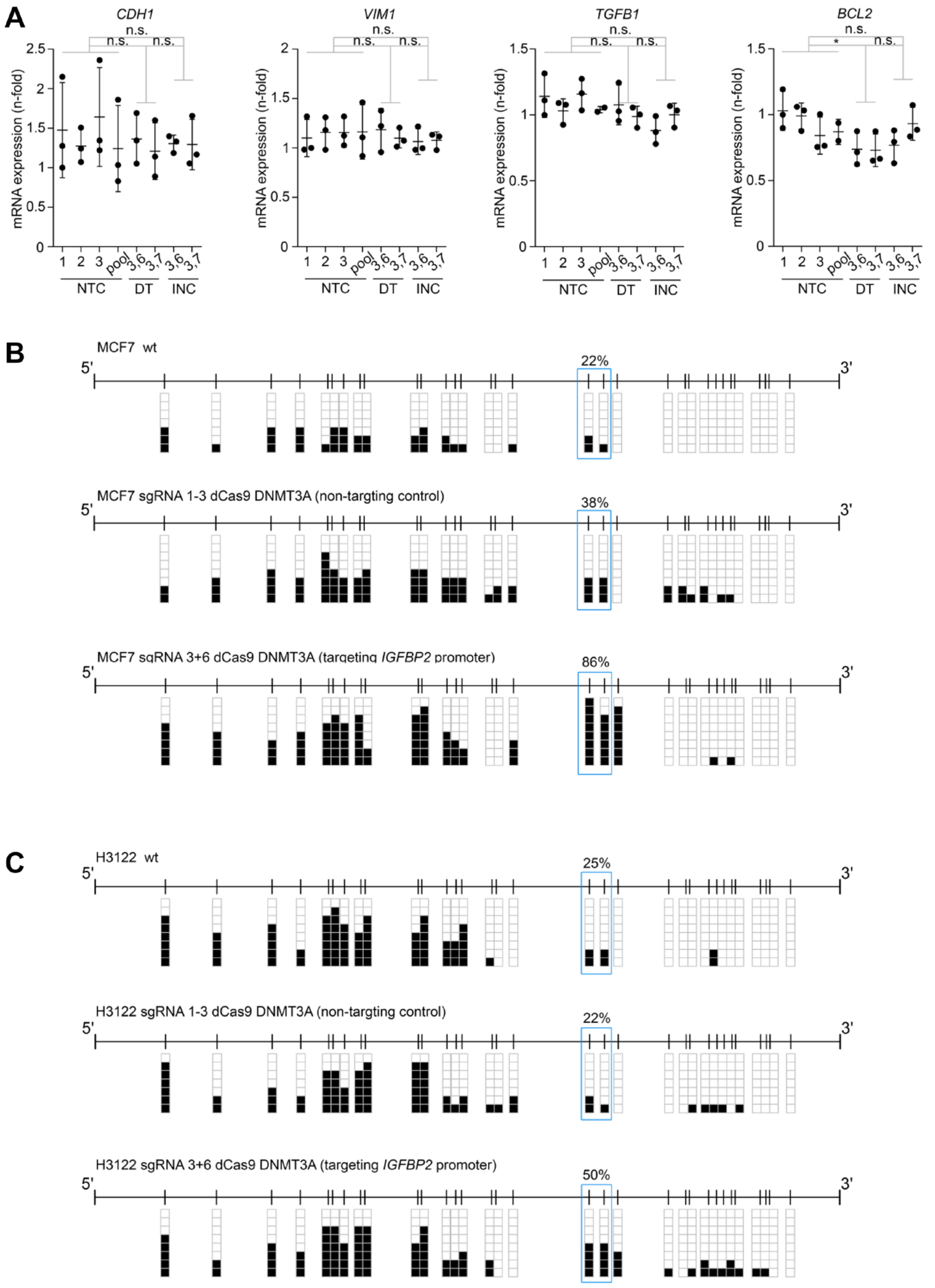

Supplementary Figure 4: (**A**) Expression data of EMT genes and apoptotic marker BCL2 in HEK293T cells targeted with dCas9/ DNMT3A constructs. Comparisons are stablished between 3 independent biological replicates [including control populations (both single or pooled control sgRNAs 1-3) and 2 independent IGFBP2 targeted populations (sgRNAs 3,6 and 3,7)]. Data is normalized using first control and housekeeping genes B2M and PPIA (error bars represent SD). Individual *p* values were obtained from Tukey's multiple comparison test. NTC: non-targeting control, DT: direct targeting, INC: DNMT3A inactive control. (**B**) Bisulfite sequencing of *IGFBP2* locus in native and dCas9/DNMT3A targeted MCF7 cells (old batch). (**C**) Bisulfite sequencing of *IGFBP2* locus in native and dCas9/DNMT3A targeted H3122 cells. Single sgRNA guides tethering positions are indicated by colored arrows. Bisulfite genomic sequencing was carried out in 7-8 individual clones. The presence of a methylated or unmethylated cytosine is indicated by a black or white square, respectively. CpGs sites of interest are highlighted with a blue squared and the percentage of methylation is indicated.

A

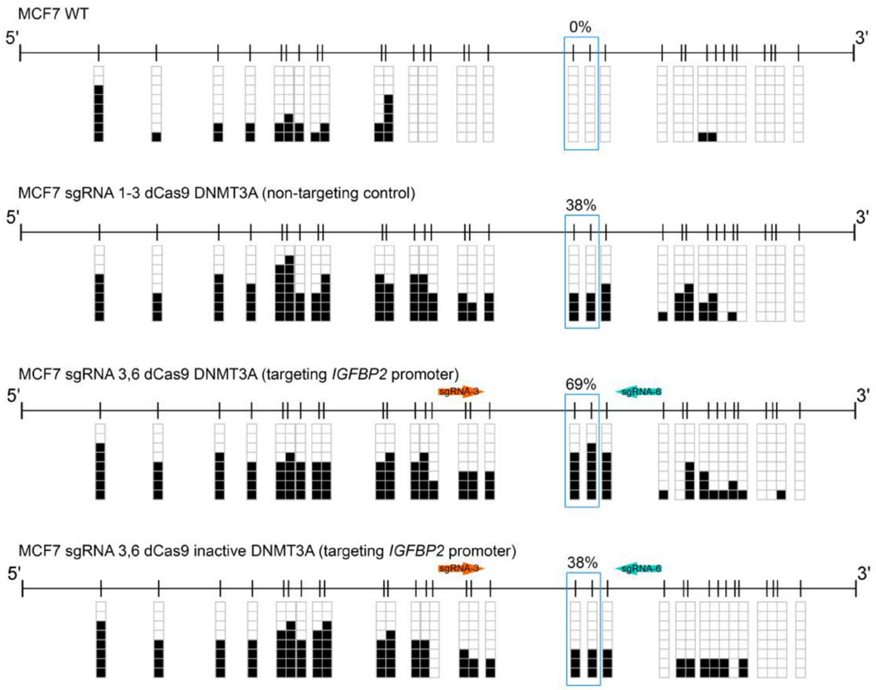

B

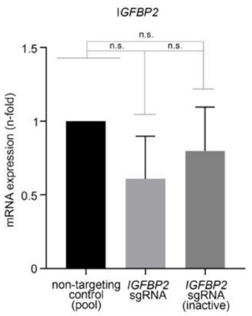

C

Marker Analysis

Marker Name	1		2		3	
	Sample Results	NCI-H3122 (JCNI 102:1310-21 (2010))	Sample Results	MCF-7 (Nature 520:307-311, 2015)	Sample Results	MCF-7 (Nature 520:307-311, 2015)
AMEL	X	X	X	X	X	X
CSF1PO	11, 12	11, 12	10, 11	10	10	10
D13S317	10, 12	10, 12	11	11	11	11
D16S539	11, 12	11, 12	11, 12	11, 12	11, 12	11, 12
D18S51	13, 16	NA	14	14	14	14
D21S11	28, 29	NA	30	30	30	30
D3S1358	16	NA	16	16	16	16
D5S818	11, 12	11, 12	11, 12	11, 12	11, 12	11, 12
D7S820	8, 12	8, 12	8, 9	8, 9	8, 9	8, 9
D8S1179	13, 15	NA	10, 14	10, 14	10, 14	10, 14
FGA	18, 21	NA	23, 24, 25	23, 25	23, 24, 25	23, 25
Penta_D	10, 13	NA	12	12	12	12
Penta_E	12	NA	7, 12	7, 12	7, 12	7, 12
TH01	7, 9.3	7, 9.3	6	6	6	6
TPOX	10	10	9, 12	9, 12	9, 12	9, 12
vWA	16	16	14, 15	14, 15	14, 15	14, 15
Identity Match	100%		> 80%		> 80%	

Supplementary Figure 5: (**A**) Bisulfite sequencing of *IGFBP2* locus in native and dCas9/DNMT3A targeted MCF7 cells (new batch). Single sgRNA guides tethering positions are indicated by colored arrows. Bisulfite genomic sequencing was carried out in 7-8 individual clones. The presence of a methylated or unmethylated cytosine is indicated by a black or white square, respectively. CpGs sites of interest are highlighted with a blue squared and the percentage of methylation is indicated. (**B**) Expression data of *IGFBP2* gene in MCF7 cells (new batch) targeted with dCas9/DNMT3A constructs. Comparisons are stablished between 3 independent biological replicates. Data is normalized using first control and housekeeping genes *B2M* and *PPIA* (error bars represent SD). Statistical analyses were performed using one sample *t* test after log2 data transformation. Two–tailed *p* values ≤ 0.05, ≤ 0.01, or ≤ 0.001 are considered statistically significant and indicated by an asterisk (*, **, or *** respectively). (**C**) Cell check analysis (IDEXX) of the cell lines used in this study.

Supplementary Material 1: Vectors used in this study. See Supplementary Material 1

Supplementary Table 1A: sgRNA guide used in the study

sgRNA guide	Sequence	Targeted strand
sgRNA-Control-1	ACGGAGGCTAAGCGTCGCAA	
sgRNA-Control-2	CAGGAGTCGCCGATACGCGT	
sgRNA-Control-3	GAATCGACCGACACTAATGT	
sgRNA-IGFBP2-1	ATACGGATAACTCGCGGGTT	-
sgRNA-IGFBP2-2	GGAGAATACGGATAACTCGC	-
sgRNA-IGFBP2-3	AGTTATCCGTATTCTCCTTC	+
sgRNA-IGFBP2-4	CTCCTTCAGGAGTCATAGTC	+
sgRNA-IGFBP2-5	ATACGGATAACTCGCGGGTT	-
sgRNA-IGFBP2-6	CCGCGTTGGCTGCGGGCTCT	-
sgRNA-IGFBP2-7	CCGCTCGCCCACCTCCGCGT	-
sgRNA-BACH2-8	AATGTAGCGATTGAGAGTGT	+
sgRNA-BACH2-3	CGCCGAATGTGTGCTCTCCC	-

Supplementary Table 1B: Pyrosequencing primers used in the study

Pyrosequencing primer	Sequence
IGFBP2-F	TTGAATTGAGAGTAGATAAAAGTA
IGFBP2-R[biotinylated]	GGAAGAGTAGGGAATTTTTAG
IGFBP2-seq	GAGTTATAGTTAGGTTAGAAGA
BACH2-F (DMR1)	GTAAAGTTATTGTGAATGGGGAG
BACH2-R[biotinylated] (DMR1)	ACTACTACTACTACTACTACTAAAAC
BACH2-seq (DMR1)	GATTGAGAGTGTGGG
BACH2-F (DMR2)	GTTTTTATGGTATTTTTTAGGAA
BACH2-R[biotinylated] (DMR2)	TCCCTCTACTATTCCAAA
BACH2-seq (DMR2)	GAAGGGGGTGGGAGT

Supplementary Table 1C: BS primers used in the study

BS primer	Sequence
BS-KDM4B-F	AGTTTATTTTTTGTGTTTGGGG
BS-KDM4B-R	AAACACCTAACACCTTCTTCATC
BS-IGFBP2-F	GGGTGATATTTTAGGATGGAAG
BS-IGFBP2-R	TTAAAAACCACTCCCTTCC

Supplementary Table 1D: MSP primers used in the study

MSP primer	Sequence
MSP_M_IGFBP2-F	TTAGAAGAGTGCGGAGGGAC
MSP_M_IGFBP2-R	GACAAATAAATACGCGCACG
MSP_UN_IGFBP2-F	AGGTTAGAAGAGTGTGGAGGGAT
MSP_UN_IGFBP2-R	AACAAATAAATACACACACACCC

Supplementary Table 1E: qPCR primers used in the study

qPCR primer	Sequence
IGFBP2-all isoforms-F	CCTCTGGAGCACCTCTACTC
IGFBP2-all isoforms-R	GAGACATCTTGCACTGTTTGAG
IGFBP2-isoform1-F	GGGCACTTGTGAGAAGCG
IGFBP2-isoform1-R	TGAGTGGTCATCGCCATTGTC

EMT and apoptotic related gene primers

qRT_CDH1_F	GTCCTGGGCAGAGTGAATTT
qRT_CDH1_R	GACCAAGAAATGGATCTGTGG
qRT_VIM_F	CGAGGAGAGCAGGATTTCTC
qRT_VIM_R	GGTATCAACCAGAGGGAGTGA
qPCR_TGFB1-F	TACCTGAACCCGTGTTGCTCTC
qPCR_TGFB1-R	GTTGCTGAGGTATCGCCAGGAA
qRT_BCL2-F	ATGCTGTACTTAAAAAATACAACATCAC
qRT_BCL2-R	GGAACACTTGATTCTGGTGTTT

CRISPR/dCas9 off-target gene primers

qRT_VILL-F	CTGGTGATCTTCCAGGAGAGA
qRT_VILL-R	CCTTGCACTTGGAAAAGCC
qRT_SHC2-F	CCATCAACCAGAGAGCCT
qRT_SHC2-R	ATTGTGCTCCAAAGAGTCCT
qRT_KDM4B-F	CTCAAGTGACGAGGAGGC
qRT_KDM4B-R	TGGCAGTAGGGGTAGAAGA
qRT_FAM184B-F	GCCCTGCTGCTAGAGTCG
qRT_FAM184B-R	CTCTGTGGCGATTATGTGAT
qRT_QSOX2-F	AACAACAAGATGCGGATTTC
qRT_QSOX2-R	CAGTTTCCAGAGAGAACACG
qRT_SPATC1L-F	GGAGCTACTACCTCAATGAGATC
qRT_SPATC1L-R	GAGGTCTGCTCGATCTTCTC
qRT_FBRSL1-F	GGAGCAGGAACGGGACC
qRT_FBRSL1-R	CTCCTTGACCCGAGGTT
qRT_KEAP1-F	CACAACAGTGTGGAGAGGTATG
qRT_KEAP1-R	ACTCGTTCCTCTCTGGGTAG

qRT_GATA4-F	TCCGTGTCCCAGACGTT
qRT_GATA4-R	GTGATTATGTCCCCGTGA
qRT_TERT_F	AGCATTGGAATCAGACAGC
qRT_TERT-R	ACGACGTAGTCCATGTTCAC
qRT_CD24-F	CTCCTACCCACGCAGATTTA
qRT_CD24-R	GTGAGACCACGAAGAGACTG
qRT_MANSC1-F	ACAAAAAACATATCAGGGGACA
qRT_MANSC1-R	TTCTGGTCAAAGATGGAAAAT
qRT_ATAD3A/B-F	TGGAGCACTCGCGTTATG
qRT_ATAD3A/B-R	TCTTTGAGCTTGGACTGTTG

Housekeeping genes

B2M-F	CCTGAATTGCTATGTGTCTGGGTTTC
B2M-R	CTCCATGATGCTGCTTACATGTCTCG
PPIA-F	ATGGTCAACCCCACCGTGT
PPIA-R	TCTGCTGTCTTTGGGACCTTG

Supplementary Table 1F: Antibodies

Name	Reference	Company	Application	Dilution
Anti-IGFBP2	3922	Cell Signaling	WB	1/1000
α-Tubulin	2144	Cell Signaling	WB	1/1000
Goat anti Rabbit IgG HRP	170-6515	Biorad	WB	1/3000

Supplementary Table 2A: CRISPR/dCas9 off target gene candidates for sgRNA3. See Supplementary Table 2A

Supplementary Table 2B: CRISPR/dCas9 off target gene candidates for sgRNA6. See Supplementary Table 2B

Supplementary Video 1: Confocal microscopy timelapse of MCF7 (old batch) clone 1 (transfected with CRISPR/dCas9 DNMT3A and control guides sgRNA1-3 as a pool). See Supplementary Video 1

Supplementary Video 2: Confocal microscopy timelapse of MCF7 (old batch) clone 2 (transfected with CRISPR/dCas9 DNMT3A and IGFBP2 guides sgRNA3,6 as a pool). See Supplementary Video 2

Supplementary Video 3: Confocal microscopy timelapse of MCF7 (old batch) clone 5 (transfected with CRISPR/dCas9 DNMT3A and IGFBP2 guides sgRNA3,6 as a pool). See Supplementary Video 3

Supplementary Video 4: Confocal microscopy timelapse of MCF7 (old batch) clone 10 (transfected with CRISPR/dCas9 DNMT3A and IGFBP2 guides sgRNA3,6 as a pool). See Supplementary Video 4
